# Job Satisfaction and Associated Factors among Medical Staff in Tertiary Public Hospitals: Results from a National Cross-Sectional Survey in China

**DOI:** 10.3390/ijerph15071528

**Published:** 2018-07-19

**Authors:** Huixuan Zhou, Xueyan Han, Juan Zhang, Jing Sun, Linlin Hu, Guangyu Hu, Shichao Wu, Pengyu Zhao, Feng Jiang, Yuanli Liu

**Affiliations:** School of Public Health, Chinese Academy of Medical Sciences & Peking Union Medical College, 5 Dongdansantiao, Dongcheng District, Beijing 100730, China; chouhuixuan@live.cn (H.Z.); hanxueyan611@163.com (X.H.); ncd_juan@163.com (J.Z.); sunjing@sph.pumc.edu.cn (J.S.); lynn0319@163.com (L.H.); hugy@sph.pumc.edu.cn (G.H.); shixizhiwsc@foxmail.com (S.W.); james880715@163.com (P.Z.); jiangfeng108@126.com (F.J.)

**Keywords:** job satisfaction, individual characteristics, job-related factors, tertiary public hospitals, China

## Abstract

Medical staff in China’s tertiary public hospitals are responsible for providing healthcare to a considerable number of patients, and their job satisfaction needs attention. The aim of this study is to investigate the job satisfaction of medical staff in tertiary public hospitals and to explore its associated factors. Based on a national survey conducted in 2016, this study included 43,645 physicians and nurses nested in 136 tertiary public hospitals in 31 provinces of China. Multi-level logistic regression was used to examine job satisfaction and its association with individual characteristics and job-related factors. Results showed that 48.22% respondents were satisfied with their job, and they were least satisfied with their compensation. Individual characteristics including occupation, gender, education background, alcohol drinking and self-reported health status, as well as job-related factors regarding professional title, work years, income, workload, doctor-patient relationship and practice setting were found to be significantly associated with job satisfaction. Given that some of these factors may be amenable to interventions, we suggest that government and hospital administrators could take some measures to promote continuing education, improve personal health, balance workload and compensation for medical staff, in order to improve the job satisfaction of medical staff in tertiary public hospitals.

## 1. Introduction

The Chinese government announced a series of health reforms in 2009 to improve access to public health facilities and reduce the individual medical burden. As a result, the coverage of the basic insurance has expanded, and public health services have become more available. Studies also found that residents’ satisfaction of health service accessibility and medical costs has improved [1,2]. 

However, the policy focusing on improving patient services may overlook the welfare of healthcare providers. The advancement of a zero markup policy of drug sales in public hospitals since 2012 tended to reduce hospital revenue and may adversely affect the income of medical staff [3]. In order to close the gap between the ever-increasing patient volume and limited healthcare resources, the health facilities were required to increase the workload of medical staff [2,4]. A survey showed that an increased proportion of physicians and nurses felt it difficult to meet patients’ demand and suffered from medical conflicts [2]. Researchers have confirmed that the lack of benefits, heavy workload and deteriorating work environment would adversely affect the overall job satisfaction and could also develop into higher stress rating and burnout syndrome [5]. The decreased job satisfaction can be negatively linked to medical staff’s retention, the quality of healthcare they provide and patient outcomes [6,7,8,9]. Moreover, surveys also indicated that misgivings of the current Chinese medical staff also affect the enthusiasm of enrolling in medical schools [10,11,12,13,14], while China is still in need of more professional health workers [15].

Recognizing the significance of workforce job satisfaction, researchers have started to investigate job satisfaction and intention to leave among medical staff in Chinese healthcare facilities. Most of the researchers focused on medical staff of primary and secondary health facilities, partly because of their greater mobility than medical staff in tertiary hospitals [16,17,18,19,20,21,22,23,24]. In fact, medical staff in tertiary hospitals undertake the heaviest workload among those in health facilities of different levels. The 2232 tertiary public hospitals in China account for only 7.66% of the all-type health facilities yet provide 48.70% of the outpatient services and 42.50% of the inpatient care; on average, a physician in a tertiary hospital is responsible for 8.10 outpatients and 2.70 beds per day. Such a workload is higher than that of the medical staff in secondary and primary health facilities [25]. Some provincial level investigations covering various levels of health facilities showed that the job satisfaction of medical staff in tertiary hospitals was lower than that of medical staff in lower level health facilities [22,23,24]. Therefore, the job satisfaction of medical staff in tertiary hospitals needs attention. 

When explaining variances in job satisfaction, individual characteristics (such as age, gender, health status and occupation) and job-related factors (such as workload, income, practice setting and relationship with patients) of medical staff could be included [9]. Job-related factors are typically considered amenable to intervention or strategies for satisfaction improvement. Individual characteristics are generally unchangeable but can reflect the response of medical staff of different types to their job [9]. Previous researchers have indicated that some demographic factors, work hours and doctor-patient relationship are significantly associated with job satisfaction [9,16,21]. Appropriate state of health is also essential for good performance at work [26]. However, the job satisfaction of physicians and nurses in tertiary hospitals and its association with these factors need to be examined at a national level in China, especially when including some individual health factors and job-related factors together. Moreover, there are some controversial results about the association between some factors and job satisfaction in some provincial level studies. For instance, a study in Guangdong Province in China indicated that nurses, junior titles and fewer working years were positively associated with job satisfaction [16], while a study conducted in Hubei province demonstrated that job satisfaction of medical staff in public hospitals had no significant relationship with these factors [21]. 

The aim of this study is to investigate the job satisfaction of physicians and nurses in tertiary public hospitals and to explore its associated factors by a national sample, to identify some predictors of job satisfaction and to give some implications for improvement of satisfaction among physicians and nurses in tertiary public hospitals. 

## 2. Materials and Methods

### 2.1. Data

The study was based on part of the data from China’s Healthcare Improvement Initiative Survey, which was sponsored by China’s Ministry of Health (MoH, renamed as National Health and Family Planning Commission in 2014) with the purpose of improving healthcare quality and satisfaction of providers and patients in China’s tertiary public hospitals. The survey was conducted in 136 tertiary public hospitals across all 31 provinces of China in December 2016. The hospitals were purposively sampled by the MoH: among them, 43 tertiary hospitals are affiliated with the national health administration, and 93 tertiary hospitals are affiliated with the provincial government (3 in each of the 31 provinces, selected based on their year of foundation and popularity in the area). These hospitals accounted for 6.09% of all the tertiary hospitals, delivered 11.60% of the inpatient care among tertiary hospitals and hired 82,948 physicians (10.30% of all physicians in tertiary hospitals) and 124,647 nurses (10.00% of all nurses in tertiary hospitals) [25]. The physician and nurse samples were selected from the entire staff list of each hospital using a stratified random sampling method. The strata were divided based on professional titles (junior, middle and senior). The designated sample size was 150 physicians and 180 nurses per hospital. A smartphone-based online system was used to conduct the survey. Finally, 44,086 respondents including both physicians and nurses completed the online questionnaire. The response rate was 96.93% among physicians and 99.31% among nurses. 

### 2.2. Ethics Statement

The study protocol was approved by the Ethics Committee of School of Public Health, Peking Union Medical College (SPH201611CHII206). The survey of job satisfaction was anonymous. Informed consent was provided in the homepage of online survey and was obtained when participants completed the survey. 

### 2.3. Measures

Job satisfaction is the outcome measure in this study. According to the Job Satisfaction Index [27], eight items were used to reflect the attitudes of physicians and nurses towards their job: work itself, superiors, promotion, environment, compensation, colleagues, facilities and current work. 

Doctor-patient relationship was measured by medical staff’s perceptions of three statements, which were adapted from similar research [16]: the society respects the career of medical staff, patients respect medical staff, recent doctor-patient relationships have been improved. The statements of job satisfaction and doctor-patient relationship were measured by a 5-point Likert scale: strongly disagree-1, disagree-2, moderate-3, agree-4, strongly agree-5 [28]. 

Health status was measured by three items related to health and bodily pain [26,29], and a 5-point Likert scale was also used: self-rated physical health (very poor-1, poor-2, regular-3, good-4, very good-5), self-rated mental strain (very severe-1, severe-2, moderate-3, mild-4, none-5), perceived bodily pain in the previous month (very severe-1, severe-2, moderate-3, mild-4, none-5).

Exploratory factor analysis was conducted to evaluate the construct validity of the 14 items related to job satisfaction, doctor-patient relationship and health status. The results of factor loading are demonstrated in Table 1. The factor loading of each item was larger than 0.50, which indicated that the construct validity of these items was acceptable [16]. The Cronbach’s alpha value of these items across three dimensions (see Table 1) was larger than 0.70, which showed a good reliability of these items in this survey [30].

Besides, other individual characteristics including age, gender, marital status, occupation, educational background, smoking and drinking, as well as job-related factors including professional title, work years, monthly income, working hours per weekday, night shift frequency, department, hospital type and location were included in the analysis [9,17,21,31]. Details of these variables are listed in the Results section.

### 2.4. Statistical Analysis

Descriptive analyses were conducted, and job satisfaction along with individual and job-related factors of included physicians and nurses were reported. The scores of the three items related to doctor-patient relationship were treated as continuous variables. Other variables were treated categorically. Job satisfaction was transformed into binary categorical variable based on the mean score of the 8 items related to job satisfaction. Observations with a mean score higher than 3.50 were assigned to the satisfied group, and the rest were placed in the otherwise group. This cut-off point provided sufficient samples in the two groups. In this study, since respondents were nested in 136 hospitals, multi-level multiple logistic regression was used to examine the relationship between job satisfaction and factors involved in the analysis, which allowed for association across respondents within hospitals [32]. Stata 14 (StataCorpLP, College Station, TX, USA) was used for these statistical analyses.

## 3. Results

### 3.1. Description of Individual Characteristics and Job-Related Factors

After data scrubbing, 441 samples were dropped due to inconsistences and suspected logic errors (such as having work years exceed their age). Finally, 43,645 respondents were included and analyzed in this study. Their individual characteristics and job-related factors by their occupation as physician or nurse are shown in Table 2 and Table 3. Some cut-off points were selected to ensure balance in each stratum. There were 19,430 physicians and 24,215 nurses involved in analyses. They were predominantly women (79.30%) that were married (73.64%). Most nurses were female (97.72%). The median age of respondents was 32 years old. One third of them had a master’s or doctoral degree (32.78%); these were predominately physicians (96.48%). The smoking rate among respondents was 4.06%, and 22.35% of them drank alcohol more than once per month; 53.21% of them reported very poor, poor or regular physical health, 42.54% and 25.64% of them reported very severe, severe and moderate mental strain and bodily pain, respectively. Compared with physicians, nurses were more likely to report good or very good physical health, with no or mild mental strain and bodily pain.

As for the job-related factors, about half of physicians and nurses had junior professional titles (52.74%), worked over 8 h per weekday (49.39%), and 40.02% of them had a work schedule with night shift over 4 times per month. Their median work years was 7; 78.38% of them had a monthly income less than 10,000 RMB. The proportions of physicians who worked over 8 h and earned more than 10,000 RMB per month were higher than those of nurses. These physicians and nurses came from a variety of departments: internal medicine, surgery, pediatrics, emergency, medical technique, obstetrics and gynecology and other departments. The proportions of the respondents who practiced in general hospitals, Traditional Chinese Medicine (TCM) hospitals and other types of special hospitals were 44.54%, 21.89% and 33.57%, respectively. The proportions involved in hospitals located in the eastern, central and western provinces of China were 42.68%, 22.53% and 34.79%, respectively. The average scores of perceived society respects, patient respects and improvement of doctor-patient relationship were 2.97 (standard deviation, SD = 1.33), 3.19 (SD = 1.12) and 3.26 (SD = 1.25), respectively, with physicians displaying lower scores than nurses.

### 3.2. Job Satisfaction of Physicians and Nurses

Table 4 displays the mean scores of the 8 items related to job satisfaction in ascending order of the total value, as well as the overall job satisfaction and satisfied proportion. These respondents were least satisfied with compensation (mean = 3.22) and most satisfied with colleagues (mean = 4.06). In general, 48.22% of the respondents were satisfied with their work (mean = 3.52), with physicians displaying lower scores in each item and overall satisfaction than nurses. 

### 3.3. Multi-Level Logistic Regression Analysis

The fixed effects part of the multi-level multivariate logistic model demonstrated that several individual characteristics (occupation, gender, educational background, drinking, items related to health status) and job-related factors (professional title, monthly income, items related to doctor-patient relationship, working years, work hours per weekday, night shift frequency, department and hospital types) were significantly associated with the job satisfaction of the respondents in the 136 tertiary public hospitals (see Table 5). 

Specifically, nurses were 1.76 times more likely to report satisfaction with their job than physicians (OR = 1.76, *p* < 0.001). Female respondents and those with drinking habits were less likely to be satisfied with their job (OR = 0.88, *p* < 0.001; OR = 0.85, *p* < 0.001). Respondents had a bachelor’s degree and those who had master’s or doctoral degrees were more likely to be satisfied with their job satisfaction, compared to those with lower levels of education (OR = 1.17, *p* < 0.001; OR = 1.33, *p* < 0.001). Self-rated physical health was positively associated with job satisfaction (refer to very poor/poor physical health, OR = 1.21, *p* < 0.001 for regular status, OR = 1.66, *p* < 0.001 for good/very good status). Respondents reporting moderate and severe/very severe mental strain and bodily pain were less likely to be satisfied with their job than those reporting none/mild mental strain (OR = 0.67, *p* < 0.001; OR = 0.58, *p* < 0.001) and bodily pain (OR = 0.86, *p* < 0.001; OR = 0.75, *p* < 0.001). 

Regarding job-related factors, respondents with middle, associate senior and senior titles were more likely to be satisfied with their job, compared to those with a junior professional title (OR = 1.11, *p* < 0.05; OR = 1.15, *p* < 0.05; OR = 1.26, *p* < 0.001). Earning a monthly income more than 5000 RMB increased the likelihood for the respondents to report job satisfaction (OR = 1.33, *p* < 0.001 for 5000 to 9999 RMB; OR = 1.45, *p* < 0.001 for 10,000 to 20,000 RMB; OR = 1.19, *p* < 0.05 for more than 20,000 RMB). When scores of the items related to society respects, patient respects and improvement of the doctor-patient relationship increased by 1 point, the likelihood for the respondents to be satisfied with their job increased by 66%, 55% and 61%, respectively (OR = 1.66, *p* < 0.001; OR = 1.55, *p* < 0.001; OR = 1.61, *p* < 0.001). Working for more than 4 years decreased the likelihood for the respondents to feel satisfied with work (OR = 0.84, *p* < 0.001 for 4 to 7 years; OR = 0.90, *p* < 0.05 for 8 to 15 years; OR = 0.85, *p* < 0.05 for more than 15 years). Respondents working 8 to 10 h per weekday and more than 10 h per weekday were less likely to be satisfied with their job, compared with those did not work overtime (OR = 0.73, *p* < 0.001; OR = 0.62, *p* < 0.001). With night shift frequency exceeding 4 times per month, working in a department of medical techniques and special hospitals other than TCM resulted in a 17%, 14% and 26% decreased likelihood, respectively, for respondents to report satisfaction with their job, with respect to those with less night shift frequency, working in a department of internal medicine and general hospitals (OR = 0.83, *p* < 0.001; OR = 0.86, *p* < 0.001; OR = 0.74, *p* < 0.05). 

Age, marital status, smoking, location of hospitals, and departments except for medical techniques did not have a significant association with job satisfaction in this study.

## 4. Discussion

This study examined the job satisfaction among medical staff in tertiary public hospitals in China and explored its associated factors. In addition, the descriptive analysis provides a profile of the respondents. The special value of this study is that it not only focuses on medical staff in tertiary hospitals, who bear the heaviest workload among medical staff in health facilities of various levels, but it also integrates several individual characteristics and job-related factors in one model based on a national dataset. Several factors included in this model have seldom been tested in China, or controversial conclusions in different studies at provincial or hospital levels have been drawn. 

### 4.1. Profile and Job Satisfaciton of Physicians and Nurses in Teritiary Public Hospitals

Based on the descriptive analysis, 71.04% of physicians had master’s or doctoral degrees, and 69.66% of nurses had a bachelor’s or higher degree, which exceeded the proportions among physicians and nurses of all China’s health facilities (11.20% of all physicians had master’s or doctoral degrees, 16.30% of all nurses had a bachelor’s or higher degree) [25]. Less than 10% of the respondents reported poor or very poor physical health, severe or very severe metal strain and bodily pain, which demonstrated that most physicians and nurses in tertiary hospitals had moderate or good health states. About 80% of the respondents reported an average monthly income of less than 10,000 RMB (about 1580 USD); about half of them worked more than 8 h per weekday and had working schedule with more than 4 night shifts per month, which indicated a relatively low income level and heavy workload compared with medical staff in some developed countries [33,34]. At the same time, their income level is much better than Chinese primary care providers [17]. The scores of perceptions related to the doctor-patient relationship ranged from 2.97 to 3.26, which were close to the moderate scale (score 3). 

The mean score of overall job satisfaction was 3.52, slightly exceeding the moderate attitude. The satisfaction rate seems to be somewhat low; only 48.22% respondents were satisfied with their job. Compensation was their least satisfactory item, which mirrored the results in similar studies in several provinces in China as well as other countries [12,16,35,36,37], and indicated that compensation was one of the important factors that contributed to job satisfaction of medical staff. 

### 4.2. Individual Characterisctics Associated with Job Satisfaciton

Nurses were more likely to be satisfied with their job than physicians. This finding is similar to some studies at the provincial level [16,24,38]. Generally, the diagnosis and treatment work of physicians is more challenging and risky than providing nursing care [38]. In this study, the proportions of physicians who worked more than 8 h per weekday, with a high frequency of night shifts, reported poor health status and the doctor-patient relationship was poorer than those of nurses, which indicated that physicians may have heavier workload, worse physical health, more mental strain and more intense relationships with patients compared with nurses. The characteristics of physicians themselves and their job may contribute to their lower job satisfaction than that of nurses. Health facilities in China are confronted with a critical shortage of nurses, and a series of studies have focused on their job satisfaction and turnover [18,20,39,40,41]. However, the findings of this study demonstrated that the job satisfaction and wellbeing of physicians in tertiary hospitals are also in need of attention.

In this study, educational background had a positive association with job satisfaction. Tertiary hospitals generally have a high proportion of medical staff with a high education level, and promotion is competitive. A better educational background could provide more opportunities to participate in academic activities and to obtain research grants, which are critical for promotion in China’ public hospitals [16]. Thus, providing an opportunity for degree-awarding continuing education may improve the job satisfaction of physicians and nurses with a low educational background in tertiary public hospitals.

Regarding the health-related factors, drinking, self-rated mental strain and bodily pain were negatively associated with job satisfaction, whereas self-rated physical health was positively related, and smoking had no significant relationship with job satisfaction. These findings suggested that health behavior and self-rated health status were associated with job satisfaction of physicians and nurses in tertiary hospitals, and interventions such as health promotion among medical staff may improve their job satisfaction [42]. 

Research among primary care providers or medical staff in various health facilities in China indicates that gender, age and marital status have no significant relationship with job satisfaction [16,17,21]. This study, which exclusively including medical staff in tertiary hospitals, demonstrated that female gender was negatively associated with job satisfaction, which was different from the results of previous research. Given the inhomogeneity of gender composition in this sample and that the relationship between job satisfaction and gender is likely to be confounded by other unobservable factors, a conclusion may be difficult to draw [9]. However, this finding could indicate that the job satisfaction of female staff in tertiary hospitals requires attention, and the gender differences among diverse facets of job satisfaction could be explored in further studies.

### 4.3. Association of Job Satisfaction with Job-Related Factors

Mirroring the results in similar studies [9,16,21], this study indicated that long work hours and more frequent night shifts were negatively associated with job satisfaction. A higher income level and a benign doctor-patient relationship had a positive correlation with satisfaction. Apart from consistently emphasizing improvement of patient services and experience, health reforms should also consider the benefits of medical staff. Job satisfaction of medical staff not only has an impact on the well-being of the staff but also contributes to their work engagement and the quality of healthcare [12,43]. Therefore, it is important for the government and hospital administrators take some measures to refine the work schedule of medical staff in tertiary hospitals and set the compensation at an appropriate amount, especially for those of a low income level (below 5000 RMB in this study) so that their job satisfaction may be improved. Moreover, the increasing medical disputes and violence against medical staff have been deteriorating the perceptions of the doctor-patient relationship over the past decade [2,44]. Due to the significant association between the doctor-patient relationship and job satisfaction, the government should consider improving the current legal system to effectively process medical disputes, as well as prevent patient-initiated violence against medical staff. Health literacy of patients and professional ethics of medical staff need to be improved to deal with communication barriers and distrust between health providers and patients. Mass media should avoid exaggerated or false reports about adverse medical events and rebuild respect for medical staff by fair reports [45]. 

In previous studies [9,16], medical staff with longer work years were less likely to be satisfied with their job, while the trend was not clear in this study. Medical staff with higher professional titles were more satisfied with their job than those of lower title, potentially because of better allowance and greater autonomy corresponding to higher titles. The results mirrored studies conducted in Hubei and Xinjiang provinces [22,24] but were different from studies conducted in Guangdong and Beijing [16,46]. Those studies could be limited by their sampling at the provincial or hospital level, and some included medical staff from health facilities of various levels, not just tertiary hospitals. Our study, using a national sample, thus has important implications for the job satisfaction among physicians and nurses in tertiary public hospitals. Based on this diverse sample of tertiary hospitals, this study explored the relationship of departments, hospital types and location with job satisfaction and indicated that physicians and nurses in medical technique departments and special hospitals other than TCM were less likely to be satisfied with their work, and eastern, central or western locations had no significant relationship with job satisfaction. These findings suggested that the job satisfaction of medical technicians and those who work in special hospitals need to be noted. 

### 4.4. Limitations and Suggestions for Future Research

This study has several limitations. First, tertiary hospitals included in this study are the top ones with historical standing in their provinces, and their representativeness of national tertiary hospitals cannot be ascertained. However, they present typical characteristics of tertiary hospitals such as a high professional level and heavy workload of medical staff. Second, the cross-sectional study design cannot establish causal relationships between factors and job satisfaction. Third, some self-reported or subjective measures used in this study may render some data less reliable. For example, personal income may be understated, work hours and self-rated mental strain may be exaggerated. Fourth, this study did not consider possible psychological symptoms, such as burnout syndrome or other disorders among these medical staff. The association between job satisfaction and psychological symptoms was not studied.

According to the limitations and results of this study, we suggest that future research could include more tertiary hospitals or select hospitals by a random sampling method and draw a more generalized conclusion; some objective measures such as stress scales and bodily pain scale could be involved, some psychological symptoms such as burnout and depression could receive attention, and the relationship between occupational health and job satisfaction could be explored in detail in future studies. In addition, given the significant association between some factors and job satisfaction demonstrated in this study, time-based data could be collected, and perhaps some inference of causal relationships between job satisfaction and influential factors could be developed to provide some suggestions for interventions. Furthermore, in-depth research could be conducted to explore or interpret the difference in job satisfaction among medical staff of different genders, occupation or departments in both a quantitative and qualitative way.

## 5. Conclusions

This study shows that physicians and nurses in China’s tertiary public hospitals are well educated, but have relatively low income and long work hours. Less than half of them reported satisfaction with their job, and they are least satisfied with compensation. Given that these medical staff are valuable human resources for health in China and are responsible for providing services to a considerable number of patients, health reform in public hospitals should be considered. In this study, several individual characteristics including occupation, gender, education background, drinking and self-rated health status and some job-related factors regarding professional title, work years, income, workload, doctor-patient relationship, and practice setting were found to be associated with job satisfaction, and some of these factors may be amenable to interventions. Therefore, we suggest that the government and hospital administrators take measures to promote continuing education and personal health, balance workload and income and rebuild trust and respect for medical staff, thereby improving the job satisfaction among physicians and nurses in tertiary public hospitals. 

## Figures and Tables

**Table 1:** Factor loading and Cronbach’s alpha of items across three dimensions.

Items	Job Satisfaction	Doctor-Patient Relationship	Health Status
Work itself	0.80		
Superiors	0.79		
Promotion	0.78		
Environment	0.75		
Compensation	0.70		
Colleagues	0.66		
Facilities	0.65		
Current work	0.52		
The society respects the career of medical staff		0.78	
Patients respect medical staff		0.76	
Recent doctor-patient relationship has been improved		0.71	
Self-rated physical health			0.82
Self-rated mental strain			0.76
Perceived bodily pain in previous month			0.74
Cronbach’s alpha	0.90	0.82	0.74

**Table 2:** Individual characteristics of respondents, n (%).

Variables	Physicians *n* = 19,430	Nurses *n* = 24,215	Total *n* = 43,645
Gender			
Male	8408(43.64)	553(2.28)	9033(20.70)
Female	10,950(56.36)	23,662(97.72)	34,612(79.30)
Age ^1^			
<30	3440(17.70)	11,375(46.98)	14,815(33.94)
30–36	7686(39.56)	7523(31.07)	15,209(34.85)
>36	8304(42.74)	5317(21.96)	13,621(31.21)
Marital status			
Not married	3485(17.94)	8022(33.13)	11,507(26.36)
Married	15,945(82.06)	16,193(66.87)	32,138(73.64)
Educational background			
Below bachelor degree	227(1.17)	7347(30.34)	7547(17.35)
Bachelor degree	5399(27.79)	16,365(67.58)	21,764(49.87)
Master’s or doctor’s degree	13,804(71.04)	503(2.08)	14,307(32.78)
Smoking			
No ^2^	17,829(91.76)	24,046(99.30)	41,875(95.94)
Yes	1601(8.24)	169(0.70)	1770(4.06)
Drinking			
No ^3^	12,896(66.37)	20,995(86.70)	33,891(77.65)
Yes	6534(33.63)	3220(13.30)	9754(22.35)
Self-rated physical health			
Very poor/poor	1681(8.65)	1584(6.54)	3265(7.48)
Regular	9853(50.71)	10,107(41.74)	19,960(45.73)
Good/very good	7896(40.64)	12,524(51.72)	20,420(46.79)
Self-rated mental strain			
Very severe/severe	1907(9.81)	1311(5.41)	3218(7.37)
Moderate	7856(40.43)	7494(30.95)	15,350(35.17)
None/mild	9667(49.75)	15,410(63.64)	25,077(57.46)
Perceived bodily pain			
Very severe/severe	814(4.19)	933(3.85)	1747(4.00)
Moderate	4297(22.12)	5149(21.26)	9446(21.64)
None/mild	14,319(73.70)	18,133(74.88)	32,452(74.35)

^1^ The median age of the respondents is 32 years old, the inter-quartile range (IQR) is 11. ^2^ No smoking indicates that the respondent has never smoked or has quit smoking for more than six months. ^3^ No drinking indicates that the respondent drinks less than once per month.

**Table 3:** Job-related factors of respondents, n (%).

Variables	Physicians *n* = 19,430	Nurses *n* = 24,215	Total *n* = 43,645
Professional title			
Junior	6158(31.69)	16,861(69.63)	23,019(52.74)
Middle	6946(33.75)	6294(25.99)	13,240(30.34)
Associate senior	3949(20.32)	943(3.89)	4892(11.21)
Senior	2377(12.23)	117(0.48)	2494(5.71)
Working years ^1^			
<4 years	5416(27.87)	6207(25.63)	11,623(26.63)
4–7 years	4480(23.06)	6605(27.28)	11,085(25.40)
8–15 years	4586(23.60)	5822(24.04)	10,408(23.85)
>15 years	4948(25.47)	5581(23.05)	10,529(24.12)
Average monthly income			
<5000 RMB	3643(18.75)	7432(30.69)	11,075(25.38)
5000–9999 RMB	9388(48.32)	13,745(56.76)	23,133(53.00)
10,000–20,000 RMB	4985(25.66)	2514(10.38)	7499(17.18)
>20,000 RMB	1414(7.28)	524(2.16)	1938(4.44)
Average work hours per day ^2^			
<8 h	5930(30.52)	16,162(66.74)	22,092(50.62)
8–10 h	8790(45.24)	6842(28.26)	15,632(35.82)
>10 h	4710(24.24)	1211(5.00)	5921(13.57)
Night shift frequency			
0–4 times per month	11,299(58.15)	14,879(61.45)	26,178(59.98)
>4 times per month	8131(41.85)	9336(38.55)	17,467(40.02)
Department			
Internal medicine	5441(28.00)	6557(27.08)	11,998(27.49)
Surgery	5554(28.58)	7516(31.04)	13,070(29.95)
Pediatrics	1822(9.38)	2369(9.78)	4191(9.60)
Emergency	806(4.15)	1874(7.74)	2680(6.14)
Medical technique	1595(8.21)	1098(4.53)	2693(6.17)
Obstetrics and gynecology	2847(14.65)	3650(15.07)	6497(14.89)
Other departments	1365(7.03)	1151(4.75)	2516(5.76)
Hospital type			
General	8526(43.88)	10,915(45.08)	19,441(44.54)
Traditional Chinese Medicine	4319(22.23)	5235(21.62)	9554(21.89)
Other special types	6585(33.89)	8065(33.31)	14,650(33.57)
Location			
East	8358(43.02)	10,268(42.40)	18,626(42.68)
Middle	4553(23.43)	5282(21.81)	9835(22.53)
West	6519(33.55)	8665(35.78)	15,184(34.79)
The society respects the career as medical staff, mean (SD)	2.48(1.29)	3.37(1.22)	2.97(1.33)
The patients respect physicians and nurses, mean (SD)	3.02(1.12)	3.26(1.10)	3.19(1.12)
Recent doctor-patient relationship is getting better, mean (SD)	2.92(1.27)	3.54(1.17)	3.26(1.25)

^1^ The median (IQR) of work years is 7(11). ^2^ The median (IQR) of work hours is 8(2).

**Table 4:** Job satisfaction of respondents, mean (SD).

Items	Physicians *n* = 19,430	Nurses *n* = 24,215	Total *n* = 43,645
Compensation	3.01(1.10)	3.38(1.15)	3.22(1.14)
Current work	2.76(1.32)	3.66(1.23)	3.26(1.35)
Environment	3.15(1.10)	3.57(1.07)	3.38(1.10)
Facilities	3.08(1.20)	3.74(1.12)	3.45(1.20)
Promotion	3.35(1.03)	3.69(1.02)	3.54(1.04)
Work itself	3.51(1.00)	3.68(1.02)	3.60(1.01)
Superiors	3.43(1.03)	3.82(0.97)	3.65(1.02)
Colleagues	3.96(0.76)	4.14(0.78)	4.06(0.78)
Overall satisfaction *	3.28(0.80)	3.71(0.82)	3.52(0.84)
Satisfied%	35.87	58.12	48.22

* Overall satisfaction was the average of eight items of job satisfaction.

**Table 5:** Logistic regression examining individual characteristics and job-related factors associated with job satisfaction (satisfied versus otherwise).

Variables	Odds Ratio	95.0% CI (Lower)	95.0% CI (Upper)
Individual characteristics			
Occupation of nurse (ref. physician)	1.76 **	1.61	1.93
Female (ref. male)	0.88 **	0.80	0.95
Age (ref. <30)			
30–36	1.04	0.95	1.13
>36	0.98	0.86	1.12
Married (ref. not married)	0.98	0.91	1.05
Educational background (ref. below bachelor degree)			
Bachelor degree	1.17 **	1.09	1.26
Master’s or doctor’s degree	1.33 **	1.18	1.49
Smoker (ref. no)	0.97	0.84	1.12
Drinker (ref. no)	0.85 **	0.79	0.91
Self-reported physical health (ref. very poor/poor)			
Regular	1.22 **	1.08	1.37
Good/very good	1.66 **	1.45	1.89
Self-reported mental strain (ref. none/mild)			
Moderate	0.67 **	0.63	0.71
Severe/very severe	0.58 **	0.51	0.66
Perceived bodily pain (ref. none/mild)			
Moderate	0.86 **	0.80	0.91
Severe/very severe	0.75 **	0.65	0.86
Job-related factors			
Professional title (ref. junior)			
Middle	1.11 *	1.02	1.20
Associate senior	1.15 *	1.01	1.30
Senior	1.26 **	1.08	1.47
Work years (ref. <4 years)			
4–7 years	0.84 **	0.77	0.90
8–15 years	0.90 *	0.81	0.99
>15 years	0.85 *	0.74	0.96
Average monthly income (ref. < 5000 RMB)			
5000–9999 RMB	1.33 **	1.23	1.42
10,000–20,000 RMB	1.45 **	1.30	1.60
> 20,000 RMB	1.19 *	1.03	1.38
Average work hours per day (ref. < 8 h)			
8–10 h	0.73 **	0.69	0.77
>10 h	0.62 **	0.56	0.67
Night shift (ref. 0–4 times per month)			
>4 times per month	0.83 **	0.78	0.88
Department (ref. internal medicine)			
Surgery	0.96	0.90	1.03
Pediatrics	1.08	0.97	1.20
Emergency	1.01	0.90	1.13
Medical technique	0.86 *	0.77	0.96
Obstetrics and gynecology	1.06	0.96	1.17
Other departments	1.04	0.93	1.17
Hospital type (ref. general)			
Traditional Chinese Medicine	0.88	0.66	1.16
Other special types	0.74 *	0.57	0.94
Location (ref. east)			
Middle	0.81	0.61	1.06
West	0.79	0.61	1.02
The society respects the career as a medical staff	1.66 **	1.61	1.71
The patients respect physicians and nurses	1.55 **	1.50	1.61
Recent doctor-patient relationship is getting better	1.61 **	1.57	1.65

* *p* < 0.05, ** *p* < 0.001.
